# Verification of the Impact of Sports Event Service Quality and Host Destination Image on Sports Tourists’ Behavioral Intentions Through Meta-Analytic Structural Equation Modeling

**DOI:** 10.3390/bs15081019

**Published:** 2025-07-27

**Authors:** Hui Jia, Daehwan Kim, Kyungun Kim

**Affiliations:** 1 Department of Marine-Sports, Pukyong National University, Busan 48513, Republic of Korea; j18300633477@gmail.com; 2 Department of Sport Management, University of Central Missouri, Warrensburg, MO 64093, USA; kykim@ucmo.edu

**Keywords:** service quality, host destination image, satisfaction, behavioral intention, MASEM

## Abstract

Given that participating in or spectating sports events plays a vital role in enhancing individuals’ mental health, understanding the key factors that promote continued participation and attendance in sports events is of significant theoretical and practical importance within the context of sports tourism. From this perspective, the service quality of sports events and the image of the host destination have been identified as major determinants of sustained engagement among sports tourists. However, a review of the literature reveals that findings on the influence of sports event service quality and host destination image on the behavioral intentions of sports tourists have been inconsistent. Therefore, the purpose of this study is to employ a meta-analytic structural equation modeling (MASEM) approach to synthesize data from 39 independent studies comprising 16,335 participants, which were collected up to 30 September 2024, thereby providing generalizable conclusions. The results indicate that, first, host destination image is the most critical factor in enhancing visitor satisfaction. Additionally, the service quality of sports events significantly influences visitor satisfaction, which in turn impacts their future behavioral intentions. Second, tourist satisfaction fully mediates the relationship between event service quality and behavioral intentions, and it partially mediates the relationship between host destination image and behavioral intentions. Third, under the moderating effect of event scale (small scale vs. mega scale), host destination image and physical environment quality are more important in small-scale sports events than in mega-scale sports events. Furthermore, under the moderating effect of cultural context (Eastern vs. Western), service quality dimensions are more influential in Western cultural settings, whereas host destination image is more important in Eastern cultural settings. The significance of this study lies in its integration of previously disparate findings into a unified model, offering a more comprehensive understanding of the relationships among the variables. The results provide broad implications for future academic research and practical insights for sports tourism practitioners.

## 1. Introduction

Sports events, a key element in the sports industry, serve not only as an effective promotional tool for enhancing the visibility and recognition of host cities but also as a platform for promoting individuals’ subjective well-being and mental health (Fourie & Santana-Gallego, 2011). Previous studies have shown that sports events (e.g., the Olympics, the World Cup, and marathons) can stimulate the growth of the tourism industry during event periods (Knott et al., 2017) and have a lasting impact on its continued development even after the events conclude (Gursoy et al., 2017; Preuss, 2004). However, in recent years, the enthusiasm of potential host cities for bidding on sports events appears to have stalled. Due to concerns over cost–benefit ratios and poor sustainability records, fewer cities are inclined to host such events (Müller et al., 2023). In response to this downward trend, future host nations have announced intentions to scale down these events. At the same time, international organizations such as the International Olympic Committee (IOC) and the Fédération Internationale de Football Association (FIFA) have initiated reforms. For example, FIFA has proposed increasing the frequency of the FIFA Men’s World Cup in an effort to enhance the sustainability of the event’s global impact, creating new opportunities for cities and countries that aspire to host large-scale sporting events (Fletcher et al., 2020). These new hosting models no longer blindly pursue scale or economic growth. Instead, they emphasize operating within the limits of local carrying capacity by making rational use of existing infrastructure. In addition, by paying attention to the seasonal timing of events and the compatibility between event characteristics and host city types, these models aim to promote more sustainable tourism development (Duignan et al., 2022; Fourie & Santana-Gallego, 2011). Beyond these economic effects, accumulating evidence indicates that participation in or spectating sports events can enhance subjective well-being (SWB) by fostering positive emotions, social connectedness, and life satisfaction (Fredrickson, 2001; Diener, 1984). However, based on Gibson’s (1998) typology of sports tourists, the mental health benefits of participating in sports events as athletes versus spectating as audiences may differ, suggesting the need to examine these experiences separately. In the context of participation, there may be processes or practices that diminish or deprive athletes of their humanity, dignity, or individuality, and potentially harm their physical and mental well-being (Szathmári, 2025). In contrast, within the spectating context, high-quality event experiences not only foster emotional attachment between tourists and destinations but also promote positive attitudes and contribute to psychological and physical health (Koronios et al., 2019).

Consequently, sports event tourism has established itself as a prominent area within the tourism industry (Getz & Page, 2014; Weed, 2006). Notably, the behavioral intentions of sports event tourists have emerged as a significant academic topic, due to their close relationship with the sports tourism industry and the economic development of host destinations.

From this perspective, numerous prior studies have focused on the service quality of sports events and the image of host destinations to analyze their impact on the satisfaction and behavioral intentions of sports tourists regarding both the events and the host locations (Kusumah & Wahyudin, 2024; Vegara-Ferri et al., 2020). Although researchers commonly acknowledge the importance of sports event service quality and host destination image in influencing the satisfaction and behavioral intentions of sports tourists, findings regarding the effectiveness of these variables remain inconsistent. For instance, some studies have reported that the most significant factor influencing the behavioral intentions of tourists attending large-scale sports events is the image of the host destination (Vegara-Ferri et al., 2020). In contrast, other studies focusing on small-scale sports events have found that the quality of the physical environment plays a key role in shaping the re-participation intentions of sports tourists (S. M. Ma et al., 2022).

Meanwhile, the impact of sports event service quality and host destination image on behavioral intentions can vary depending on the characteristics of sports tourists. In a study conducted by Yamaguchi and Yoshida (2022) involving 434 marathon participants, the quality of the physical environment was identified as the most influential factor in their intention to recommend or revisit the host city. However, Pahrudin et al. (2024) found that, in a survey of spectators at a motorcycle Grand Prix, the quality of interaction was the most critical factor affecting their revisit intentions. Similarly, Song et al. (2024) reported that, for a marathon held in the United States, the quality of the physical environment had the greatest impact on visitors’ revisit intentions; on the other hand, for a marathon held in China, the image of the host destination was identified as the most significant factor. These findings suggest that, even for the same scale of sports event, the influence of specific factors may differ depending on the host region. In summary, the factors influencing sports tourists’ behavioral intentions exhibit varying patterns across different contexts. Notably, differences in event scale, tourist characteristics, and cultural context can lead to variations in the importance of these same factors. Therefore, the findings on the structural relationships among sports event service quality, host destination image, and behavioral intentions validated in specific contexts—and their associated academic and practical implications—are inevitably limited in their generalizability.

The aforementioned discrepancies in research findings can be attributed to several factors, including limitations in sample size, differences in research design, and the influence of cultural factors (Biscaia et al., 2023; Zhang et al., 2014). To overcome these limitations, meta-analytic structural equation modeling (MASEM) provides an effective method for integrating empirical results from various studies into a unified model, enabling the derivation of more consistent and unbiased findings. By standardizing research designs and expanding sample sizes, MASEM facilitates a more comprehensive understanding of the relationships between variables. Compared to traditional meta-analysis, MASEM offers distinct advantages, such as the ability to construct and validate complex structural models. Additionally, it enables the analysis of both direct and indirect relationships among variables and allows for the assessment of differences in their influence under diverse conditions, such as event scale, tourist characteristics, and cultural context (Steinmetz & Block, 2022). This approach can provide more universal and practical insights for both the theoretical framework of sports tourism and its application in real-world contexts.

This study aims to explore the impact of sports event service quality (physical environment quality, outcome quality, and interaction quality) and host destination image on sports tourists’ satisfaction and behavioral intentions by synthesizing findings from multiple independent studies using meta-analytic structural equation modeling (MASEM). Through this approach, this study seeks to gain a deeper understanding of the structural relationships and psychological mechanisms among the three components of service quality, host destination image, tourist satisfaction, and behavioral intentions. Additionally, this study aims to examine whether the structural relationships vary depending on event scale, tourist type, and cultural context.

## 2. Theoretical Background

### 2.1. Sport Events (Participation and Spectatorship) and Subjective Well-Being

Subjective well-being (SWB) refers to an individual’s subjective evaluation of their own life, encompassing dimensions such as life satisfaction, the experience of positive emotions (happiness), and a sense of meaning and purpose. High levels of SWB are closely associated with improved mental and physical health (Keyes et al., 2023). In the context of sports events, increasing scholarly attention has been directed toward understanding how participation in or spectatorship of sports events can influence individuals’ SWB.

Active sport participation, defined as engaging in physical activity through sport, ranges from formal competition to recreational exercise. It has long been recognized as a key contributor to enhanced well-being. Participation in physical activity helps reduce stress and improve mood, thereby alleviating symptoms of depression and anxiety, while simultaneously promoting physical health and quality of life (Keyes et al., 2023). Empirical studies have consistently reported significant positive associations between involvement in sport and physical activity and indicators of happiness and mental health (Lera-López et al., 2021). Active participation in sport provides individuals with immediate positive affect through the enjoyment of exercise and feelings of accomplishment—such as confidence gained from performance or pride in skill development. In addition, participation in team-based sports facilitates social bonding and fosters a sense of belonging, satisfying individuals’ relational needs and thereby contributing further to their SWB. In sum, active sport participants tend to experience enhanced well-being through the fulfillment of both physical and psychological needs (Keyes et al., 2023).

Sport spectatorship, whether through live attendance or media consumption, was overlooked in discussions of well-being. However, recent studies have highlighted its positive psychological impacts. For example, J. Kim et al. (2017) conducted a study with university students comparing levels of well-being before and after watching a sports event. They found that the hedonic enjoyment, emotional meaning, and social connection experienced during spectating significantly enhanced participants’ well-being. Similarly, Jang et al. (2017), in their analysis of professional sport spectators, demonstrated that fans with strong team identification experienced greater happiness following a team victory. More recently, Kinoshita et al. (2024) employed a multi-method approach to investigate the causal relationship between sport spectatorship and SWB. Across all three methods, they found that sport viewing positively influenced subjective well-being.

In conclusion, both active participation in and spectatorship of sports events serve as meaningful contributors to subjective well-being. From this perspective, promoting sports event participation and spectatorship may serve as an effective strategy for enhancing the well-being of individuals and communities. Accordingly, identifying the determinants that facilitate participation and attendance—and empirically verifying their influence from an integrative perspective—constitutes a significant endeavor with both academic and practical implications.

### 2.2. Event Service Quality

Service quality is closely related to customer satisfaction (Parasuraman et al., 1988). According to the expectancy disconfirmation theory proposed by Oliver (1980), customer satisfaction arises from whether the perceived service quality meets expectations. With the rapid development of the service industry, the concept of service quality has attracted significant attention from many scholars, and the expectancy disconfirmation theory has become one of the major theoretical foundations in service research. Parasuraman et al. (1988) developed the classic service quality model (SERVQUAL), which consists of five core dimensions: reliability, responsiveness, assurance, empathy, and tangibles. This model assumes that service quality is determined by the gap between customer expectations and perceptions.

While the SERVQUAL model is regarded as a pioneering tool in service quality research, several issues have emerged in its practical application. For example, “expectations” are difficult to capture accurately because they can change dynamically throughout the service experience. Therefore, some researchers argue that when measuring service quality, the focus should be on “perceived service quality” rather than “expectations” (Teas, 1993; Cronin & Taylor, 1992).

Cronin and Taylor (1992) proposed the SERVPERF model. This model uses the same measurement dimensions as the SERVQUAL model but simplifies the measurement approach, overcoming the uncertainty of the expectation measurement in the SERVQUAL model and focusing solely on customers’ perceptions of actual service performance. Grönroos (1984) proposed a perceived service quality model from a different perspective. This model emphasizes the service process and outcomes, dividing service quality into two key dimensions: technical quality and functional quality.

Later, Brady and Cronin (2001) supplemented Grönroos’s (1984) model by adding physical environment quality, proposing the three-component service quality model. This model consists of physical environment quality, outcome quality, and interaction quality, highlighting the impact of the physical environment and atmosphere of the service setting on customer perceptions. The model provides a more systematic and comprehensive framework for evaluating service quality and is considered particularly applicable to the sports tourism industry (S. C. Ma & Kaplanidou, 2021; Theodorakis et al., 2015; Shonk & Chelladurai, 2008).

Accordingly, this study conceptualizes sports event service quality based on the three-dimensional service quality model proposed by Brady and Cronin (2001). This approach aims to encompass various types of sports events and facilitate future comprehensive and comparative research. Specifically, the physical environment quality dimension is defined as the tangible elements within the service environment, including facilities, equipment, and atmosphere. Next, the outcome quality dimension is defined as the experiences and satisfaction that tourists gain from the sports event service, including both emotional and practical outcomes. Finally, the interaction quality dimension is defined as the quality of interaction between service providers and tourists, including factors such as staff attitude, behavior, and communication skills.

### 2.3. Host Destination Image

The decision of tourists to choose a specific destination is based on their overall impression (image) of the destination (Crompton, 1979). Sports events serve as an important means for effectively showcasing the host destination image to tourists (Chalip, 2004). From this perspective, hosting sports events not only generates direct tourism revenue but also influences the long-term image and branding of the host destination (Gursoy et al., 2017). The host destination image shapes tourists’ initial perceptions of the area and can significantly impact their behavior, decision making, satisfaction, and loyalty (Prayag & Ryan, 2012).

Crompton (1979) identified the components of the host destination image as natural scenery, cultural resources, infrastructure, and political and economic factors. Later, Baloglu and McCleary (1999) conceptualized the host destination image in two dimensions: the cognitive image and the emotional image. Specifically, the cognitive image includes tourists’ perceptions of natural landscapes and historical culture, while the emotional image encompasses tourists’ emotional experiences with the destination. Numerous studies have indicated that the host destination image can be influenced by factors such as social media and tourists’ experiences (Hwang et al., 2024; Fairley et al., 2016; Beerli & Martin, 2004). For the comprehensive analysis targeted in this study, the host destination image is defined as tourists’ overall perception and impression of the sports event destination, including culture, nature, urban infrastructure, and social factors, as conceptualized in previous research.

### 2.4. Satisfaction

In general, satisfaction is defined as an overall evaluation formed by comparing an individual’s or group’s expectations regarding a specific product, service, or experience with what they actually perceive (experience) (Westbrook & Oliver, 1991). Oliver (1980) argued that satisfaction is a cognitive or emotional response formed after consumers compare their expectations with real outcomes; furthermore, satisfaction determines whether consumers feel pleasure or disappointment. In the field of sports tourism, satisfaction is considered a key indicator for measuring the success of an event (Getz & Andersson, 2020). Crompton and Love (1995) defined satisfaction as the overall perception and evaluation that sports tourists have about their travel experience or participation in sports activities, which reflects the relationship between expectations and real experiences. Their proposed definition of satisfaction emphasizes the impact of event quality and destination experience and is frequently used in the context of sports event tourism (Kogoya et al., 2022; S. K. Kim et al., 2016). This definition of satisfaction is closely related to the theme and scope of this study. Based on this, satisfaction is further conceptualized as sports tourists’ overall evaluation of the service quality and their personal experiences following their observation of or participation in the event.

### 2.5. Behavioral Intentions

In the field of sports tourism, behavioral intention refers to the expectations and plans that tourists have regarding future participation in specific sports tourism activities (e.g., attending a game, watching an event, participating in sports leisure, etc.) and is influenced by tourists’ attitudes toward the destination or event (Gibson, 1998). Generally, previous studies have defined behavioral intention as the intention to re-participate in a game or event, the intention to recommend the event to others, or the intention to purchase event-related products (Davras & Özperçin, 2023; Milovanović et al., 2021; Biscaia et al., 2012). Revisit intention and recommendation intention are the two most commonly used indicators of behavioral intention in sports tourism research. Revisit intention refers to whether tourists plan to re-participate in a specific sports activity or event, while recommendation intention indicates their willingness to recommend that activity or event to others (Song et al., 2023; Martínez Cevallos et al., 2020). In this study, the behavioral intentions of sports tourists are conceptualized as revisit intention and recommendation intention, the most representative indicators.

### 2.6. Structural Relationships Between Event Service Quality, Satisfaction, and Behavioral Intentions

This study employs the Three-Component Model of Service Quality to examine the differential effects of various service quality dimensions on tourists’ satisfaction in the context of sports events. The multidimensional attributes of service quality (e.g., physical environment, interaction, and outcome quality) have been widely recognized as key drivers of consumer behavioral intentions (Brady & Cronin, 2001; Parasuraman et al., 1988). According to the expectancy disconfirmation theory (Oliver, 1980), service quality in sports events is closely related to tourists’ satisfaction and behavioral intentions (Shonk & Chelladurai, 2008). Service quality factors, such as game performance, on-site entertainment, and interactions with staff, directly influence overall satisfaction, and high-quality event experiences positively impact behavioral intentions (Hyun & Jordan, 2020; Xiao et al., 2020). Specifically, a well-maintained physical environment (e.g., seating, sound system, and cleanliness) enhances tourists’ perceptions of the convenience and enjoyment of the host destination, thereby increasing satisfaction (Milovanović et al., 2021). Moreover, tourists’ identification with and emotional responses to the outcomes of a sports event directly affect their satisfaction (Jeong & Kim, 2020), and thrilling games contribute to higher satisfaction and positively influence revisit intentions (Çevik & Şimşek, 2020). Additionally, high-quality interactions between event organizers, staff, and tourists strengthen positive experiences, thereby exerting a favorable influence on both satisfaction and behavioral intentions (Lin et al., 2020; Tzetzis et al., 2014). Based on these insights, this study proposes the following hypotheses:

**Hypothesis** **1a.** *The physical environment quality of sports events will have a positive effect on tourists’ satisfaction.*

**Hypothesis** **2a.** *The outcome quality of sports events will have a positive effect on tourists’ satisfaction.*

**Hypothesis** **3a.** *The interaction quality of sports events will have a positive effect on tourists’ satisfaction.*

**Hypothesis** **1b.** *Tourists’ satisfaction will mediate the effect of the physical environment quality of sports events on tourists’ future intentions.*

**Hypothesis** **2b.** *Tourists’ satisfaction will mediate the effect of the outcome quality of sports events on tourists’ future intentions.*

**Hypothesis** **3b.** *Tourists’ satisfaction will mediate the effect of the interaction quality of sports events on tourists’ future intentions.*

### 2.7. Structural Relationships Between Host Destination Image, Satisfaction, and Behavioral Intentions

Destination image is a key factor influencing tourists’ decision making (Crompton, 1979). According to the expectancy disconfirmation theory (Oliver, 1980), the image of the host destination (e.g., safety and attractiveness) constitutes part of tourists’ expectations and subsequently affects their satisfaction (Govindarajo & Khen, 2020; Chen & Tsai, 2007). In the context of sports event tourism, tourists’ overall impression of the event destination directly influences their satisfaction, and high satisfaction positively impacts their behavioral intentions (Hyun & Jordan, 2020). For tourism-based events, an improved host destination image enhances tourists’ satisfaction, which in turn fosters their intention to participate (H. W. Lee et al., 2014). In participatory events such as marathons or triathlons, participants’ perceptions of the host destination image are particularly important. Specifically, factors such as infrastructure, transportation convenience, climate, and safety directly influence participants’ decision making and event experiences (Song et al., 2024; Xiao et al., 2020). Moreover, participants’ satisfaction serves as a key determinant of whether they will continue to participate in the same event or recommend it to others (Plunkett & Brooks, 2018). In summary, the following hypotheses are proposed:

**Hypothesis** **4a.** *Host destination image will have a positive effect on tourists’ satisfaction.*

**Hypothesis** **4b.** *Tourists’ satisfaction will mediate the effect of host destination image on tourists’ future intentions.*

**Hypothesis** **5.** *Tourists’ satisfaction will have a positive effect on behavioral intentions.*

### 2.8. Moderating Effects of Event Scale (Large Scale/Small Scale), Tourist Type (Spectator/Athlete), and Cultural Context (Eastern/Western)

Large-scale sports events are characterized by high visibility, complex organizational structures, and significant resource investment (Getz, 2008). Tourists attending such events tend to expect higher quality in outcome factors (e.g., game level) (Wang et al., 2021; Theodorakis et al., 2013). On the other hand, tourists at small-scale events primarily evaluate the value of the event from emotional and social perspectives, rather than focusing solely on the level of the game (Fleshman & Kaplanidou, 2023). Therefore, factors such as interaction quality and the host destination’s cultural, natural, and economic elements influence tourists’ satisfaction at such events (Chalip, 2006). This suggests that the evaluation criteria for sports events vary depending on the event scale.

Additionally, while the quality of sports events significantly affects the satisfaction of both athletes and spectators, the specific aspects they focus on may differ. Athletic tourists are more concerned with factors such as the competitiveness of the game, organizational structure, and facilities (Xiao et al., 2020), whereas spectators place greater emphasis on entertainment value, venue convenience, and the event atmosphere (Wang et al., 2021). According to Fernández-Martínez et al. (2021), physical facilities and interaction quality had a greater impact on spectator satisfaction than outcome quality (e.g., game level and game results). However, a 2022 study on athletic tourists showed that outcome quality had a greater impact on satisfaction than physical facilities and interaction quality (Fernández-Martínez et al., 2022). These findings suggest that the criteria for satisfaction vary depending on the type of sports tourist (athlete vs. spectator).

Lastly, special elements provided by the host destination, such as cultural heritage sites or theme parks, play an important role in attracting tourists and extending their stays (Malchrowicz-Mosko & Munsters, 2018). Eastern cultures prefer collectivism, while Western cultures favor individualism (Hofstede, 2001). These cultural values are macro factors that influence how tourists perceive service quality (S. C. Ma & Kaplanidou, 2021). In a study by Song et al. (2024), physical environment quality had a significant impact on destination image and behavioral intention for an event held in Xiamen, China. However, at the same type of event in Chicago, USA, physical environment quality had no significant effect on behavioral intentions (Song et al., 2024). This can be interpreted as a result of cultural backgrounds influencing decision making (Ramires et al., 2018). Furthermore, racial and cultural differences also affect individuals’ perceptions of their destinations (Tsai et al., 2002). In summary, the criteria for satisfaction with an event may vary depending on cultural context. The following hypotheses are proposed:

**Hypothesis** **6a.** *Event scale (large scale/small scale) will moderate the impact of event service quality on satisfaction.*

**Hypothesis** **6b.** *Event scale (large scale/small scale) will moderate the impact of destination image on satisfaction.*

**Hypothesis** **7a.** *Tourist type (spectator/athlete) will moderate the impact of event service quality on satisfaction.*

**Hypothesis** **7b.** *Tourist type (spectator/athlete) will moderate the impact of destination image on satisfaction.*

**Hypothesis** **8a.** *Cultural context (Eastern/Western) will moderate the impact of event service quality on satisfaction.*

**Hypothesis** **8b.** *Cultural context (Eastern/Western) will moderate the impact of destination image on satisfaction.*

**Figure 1:**
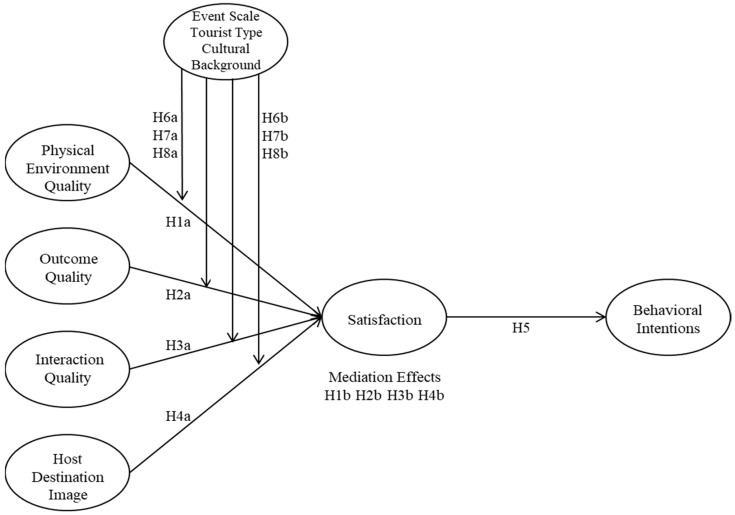
Hypothesized structural equation model.

The structural equation model for this study, grounded in the aforementioned hypotheses, is illustrated in Figure 1.

## 3. Methods

### 3.1. Meta-Analytic Structural Equation Modeling (MASEM)

Meta-analytic structural equation modeling (MASEM) is a statistical method used to integrate the effect sizes of multiple independent studies and build and validate causal relationships between variables. MASEM not only enables quantification of the overall effect size but also allows for the analysis of paths and structural relationships between latent variables. This study utilized the “metaSEM” package in the open-source integrated development environment RStudio (version 2024.09.1+394) to perform the MASEM analysis, with Pearson’s correlation coefficient (r) set as the effect size. The key advantage of using Pearson’s correlation coefficient (r) is that most original studies directly report correlation coefficients, making extraction and integration easier. Even if r is not directly reported in a study, it can be derived from other effect sizes, such as regression coefficients or mean differences. Additionally, r has a high compatibility with the key data (the correlation matrix) required for structural equation modeling.

### 3.2. Data Sources

English literature databases (e.g., Web of Science and Emerald) are primarily centered on Western countries. This study selects Korean-language databases as representatives of Eastern countries, contributing to a global perspective and balancing the weight of Eastern and Western research. Korea was chosen as the representative of Eastern countries for several reasons. First, as a prominent host of major sporting events, Korea has organized several large-scale events in recent years, including the Summer Olympics, the Winter Olympics, and the FIFA World Cup. Korea also regularly hosts smaller-scale events, such as marathons and ball games. These events provide a rich data source for studying the behavioral intentions of sports tourists. Second, Korea has one of the fastest-growing economies in Asia and shares a long history and cultural exchanges with neighboring countries such as China and Japan. As a core member of the East Asian cultural sphere, Korea offers valuable insights into the region’s cultural and historical context. Third, Korean academic databases (e.g., RISS and KISS) contain extensive research related to sports events and tourists’ behavioral intentions, providing critical resources for capturing the unique sociocultural, psychological, and behavioral patterns of Eastern countries. Furthermore, Korean databases are more systematic, accessible, and transparent than those of other Eastern countries, making them easier to utilize.

The literature for this study was collected from English research databases (Web of Science, Emerald, and Science Direct) and Korean research databases (National Assembly Library [http://dl.nanet.go.kr], Research Information Service System [RISS] [http://www.riss.kr], and Korean Studies Information Service System [KISS] [http://kiss.kstudy.com]). The search period was up to 30 September 2024. In the Korean research databases, keywords such as “sports event,” “sports event,” “mega event,” “mega sports event,” “major sports event,” “small-scale sports event,” “minor sports event,” “behavioral intention,” “revisit intention,” “recommendation intention,” and “future participation intention” were cross-combined for the search. In the English research databases, the search was conducted using the same set of keywords, and a total of 862 documents were retrieved. After these were entered into EndNote and duplicates were removed, 809 documents were ultimately secured. Below is an example of the search query used in the English databases: ((TS = (sport event)) OR TS = (sports event)) OR TS = (sporting event)) OR TS = (mega-event)) OR TS = (mega sport event)) OR TS = (major sport event)) OR TS = (small-scale sport event)) OR TS = (minor sport event)) AND TS = (behavioral intention)) OR TS = (intention)) OR TS = (revisit intention)) OR TS = (intention to revisit)) OR TS = (repeat visitation)) OR TS = (repeated attendance)) OR TS = (intention to return)) OR TS = (returning visitor intention)) OR TS = (WOM intention)) OR TS = (recommendation intention)) OR TS = (intention to recommend)) OR TS = (willingness to spread word-of-mouth)) OR TS = (participation intention)) OR TS = (engagement intention)) OR TS = (attend future events intention)) OR TS = (intention to participate in events)) OR TS = (future participation intention)) OR TS = (event engagement intention)) OR TS = (support intention)) OR TS = (intention to support team)) OR TS = (fan engagement)) OR TS = (team support intention)).

### 3.3. Literature Selection

Based on the recommendations of the PRISMA statement (Page et al., 2021), the following inclusion criteria were adopted: (1) the research topic must be related to the behavioral intentions of sports tourists; (2) the study must be published in peer-reviewed journals, ensuring that it is supported by comprehensive and high-quality evidence, leading to more reliable conclusions; (3) the study must be written in English, Korean, or Spanish. The initial search strategy was limited to English and Korean studies to align with the language proficiency of the research team. However, during the full-text screening stage, two Spanish-language studies (e.g., Ferri et al., 2021) were identified as providing authentic localized data, such as service interaction quality in sporting events held in the European region. To ensure the comprehensiveness of the evidence and reduce language bias (Walpole, 2019), the research team decided to expand the inclusion criteria to accept Spanish-language studies and verified the consistency of key terms through back-translation (e.g., ‘revisit intention’ corresponding to ‘intención de revisita’). Consequently, the final inclusion criteria were revised to include empirical studies published in English, Korean, or Spanish; (4) the study must be quantitative; (5) the data type must be related to Pearson’s correlation coefficient (r); (6) the sample size information must be explicitly stated; (7) the type and location of the event must be clearly specified; and (8) the full text of the study must be accessible.

**Figure 2:**
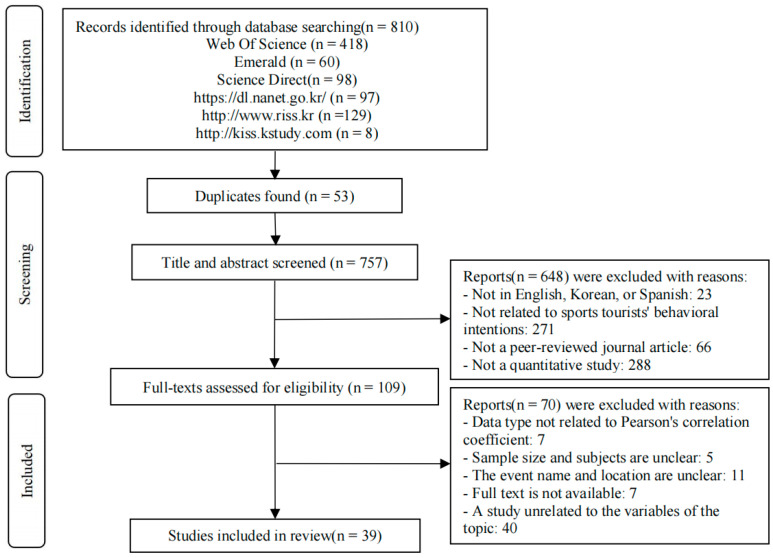
Flow chart of literature search (adapted from Page et al., 2021).

The literature screening process was conducted by three researchers, all of whom were required to have a clear understanding of the research topic and objectives. Two researchers independently screened all the retrieved studies, and in cases of disagreement, a third researcher participated in discussions to determine whether to include the study. The initial screening retrieved a total of 810 articles. In the second round, 53 duplicate articles were removed using EndNote (version 20.6) software, resulting in 757 articles. In the third round, two researchers independently reviewed the titles and abstracts of all articles and excluded 648 articles that did not meet the criteria for the research topic, language, publication type, or study type, leaving 109 articles. In the fourth round, a full-text review was conducted, and 70 articles that did not meet the requirements for data type, sample size, event information, related variables, or article quality were excluded. Ultimately, 39 articles were included, with the specific process illustrated in Figure 2.

### 3.4. Information Coding

The purpose of information coding is to standardize data on various variables and research outcomes examined in the literature, making subsequent analysis easier. Each study was assigned a unique identification number, and specific coding information included the source of the paper, first author, publication year, country, sports event scale, sample size (n), variables, and correlation coefficient (r). Ultimately, a total of 16,335 participants were included across 39 papers. To ensure consistency, objectivity, and accuracy of the coding results, this process was carried out independently by two researchers. A third researcher compared the results, and any disputed information was jointly discussed to reach a final decision. The original relevant information of the included studies is shown in Appendix A, and the relevant details are shown in Table 1.

### 3.5. Publication Bias and Heterogeneity Test

Publication bias refers to the tendency for positive or significant research results to be published more frequently than negative or non-significant findings in the academic publishing process. This bias can make the results of meta-analyses overly optimistic. Common methods for testing publication bias include funnel plots and Egger’s test (Rosenthal & DiMatteo, 2001). A funnel plot is a scatter plot where the x-axis represents the effect size (in this study, the correlation coefficient r), and the y-axis represents the sample size. In the absence of publication bias, the funnel plot will show a symmetrical distribution. Egger’s test quantifies the symmetry of the funnel plot through regression analysis to assess whether the plot exhibits a significant slope, which indicates publication bias. If the funnel plot is symmetrical and Egger’s test shows *p* > 0.05, publication bias is considered absent.

The heterogeneity test is used to determine whether there are significant differences between the data collected in a study (Sutton, 2009). Based on the results of the heterogeneity test, an appropriate analysis model is selected. In this study, I^2^ statistics (I-squared) and Q statistics (Cochran’s Q Test) were used to test for heterogeneity between studies. The I^2^ statistic is an indicator that intuitively quantifies heterogeneity between studies, with higher I^2^ values indicating greater heterogeneity. Additionally, if the Q statistic has *p* < 0.05, it indicates significant heterogeneity between studies; in this case, a random effects model should be used for data analysis. Finally, two-tailed test Z-values and *p*-values are used to assess the significance of the effects of each path.

### 3.6. Mediation and Moderation Effects

This study employed the likelihood-based method to estimate mediation effects within the hypothesized model (Karakose et al., 2025; Oort & Jak, 2016). The likelihood-based method was chosen because it provides more stable and precise parameter estimation in large samples. As the sample size increases, the standard error of parameter estimation generally decreases, thereby improving the accuracy of estimation and the reliability of model fit (Landis, 2013).

The significance of moderation effects was evaluated using the chi-square difference test. This method, widely used in structural equation modelling (SEM), determines the presence of moderation effects by comparing the chi-square values between a freely estimated model and a constrained model (Tehrani & Yamini, 2022; Jak & Cheung, 2020).

## 4. Results

### 4.1. Meta-Analysis Results

This study integrated a total of 39 relevant studies for meta-analysis, all of which were cross-sectional studies, with a combined total sample size of 16,335 participants. Table 2 presents the effect sizes (fixed effects model, random effects model, and 95% confidence intervals), heterogeneity, and publication bias across 15 variable groups. The effect size for each path was measured using correlation coefficients, and the statistical significance of the effects was tested using Z-values and *p*-values. Heterogeneity across studies was assessed using the Q statistic and I^2^ values, while publication bias was evaluated based on the symmetry of funnel plots and the *p*-values from Egger’s test.

First, all effect sizes were positive, and their 95% confidence intervals did not include zero. The Z-values exceeded the critical threshold of 1.96 for all variable groups (*p* < 0.001), indicating that the relationships between variables were statistically significant. Second, the data in this study were derived from the literature encompassing various types of sporting events, cultural contexts, and tourist categories. Given the substantial differences in the research settings of the included studies, it is appropriate to prioritize the use of a random effects model for analysis (Barili et al., 2018). In addition, the subsequent examination of moderating effects partially explained the structural sources of heterogeneity. The results showed that the I^2^ values for all variable combinations exceeded 75% (*p* < 0.001), suggesting significant heterogeneity across studies. Therefore, the random effects model was deemed appropriate for data analysis in this study. Third, the funnel plots for all variable groups were symmetrical, and Egger’s test results showed no evidence of publication bias (*p* > 0.05). This suggests that the estimated effect sizes in this study are comprehensive and reliable, with a low likelihood of bias arising from unpublished studies, particularly those with non-significant results. The correlation coefficients from the random effects model showed moderate to high correlations between all variables (Cohen, 1988). The variable most strongly correlated with satisfaction (SA) was interaction quality (r = 0.554; CI = 0.421–0.663; *p* < 0.001), while the variable most strongly correlated with behavioral intention was destination image (DI) (r = 0.520; CI = 0.446–0.587; *p* < 0.001). Table 3 presents detailed correlation coefficients.

### 4.2. Structural Equation Modeling (SEM) Analysis Results

Table 4 presents the model fit indices and path coefficients. Overall, the model demonstrated an acceptable model fit to the integrated data (RMSEA = 0.016, SRMR = 0.046, TLI = 0.978, and CFI = 0.994) (Hu & Bentler, 1999). All path coefficients were statistically significant (*p* < 0.05), and the confidence intervals did not include zero.

According to Table 4, the three sub-dimensions of service quality in sports events, namely physical environment quality (Est = 0.206, *p* < 0.05), outcome quality (Est = 0.207, *p* < 0.05), and interaction quality (Est = 0.198, *p* < 0.05), had significant positive effects on satisfaction, with nearly identical effect sizes. In addition, the host destination image (Est = 0.310, *p* < 0.05) had a significant positive influence on satisfaction, showing the strongest effect among the exogenous variables. Finally, satisfaction (Est = 0.758, *p* < 0.05) had a significant positive effect on behavioral intention. Overall, H1a, H2a, H3a, H4a, and H5 were all supported.

### 4.3. Mediating Effects of Satisfaction

The mediating effects of satisfaction were estimated using the likelihood-based method. Table 5 presents the results. Specifically, the four indirect paths involving physical environment quality, outcome quality, interaction quality, and destination image through satisfaction to behavioral intention had the following estimates: physical environment quality to satisfaction to behavioral intention (Est = 0.156, 95% CI [0.078, 0.230]), outcome quality to satisfaction to behavioral intention (Est = 0.157, 95% CI [0.057, 0.251]), interaction quality to satisfaction to behavioral intention (Est = 0.150, 95% CI [0.041, 0.249]), and destination image to satisfaction to behavioral intention (Est = 0.235, 95% CI [0.168, 0.298]). All estimates were positive, and their 95% confidence intervals did not include zero, indicating that these indirect effects were statistically significant.

Meanwhile, the direct paths from physical environment quality, outcome quality, and interaction quality to behavioral intention had the following estimates: physical environment quality to behavioral intention (Est = 0.074, 95% CI [−0.049, 0.190]), outcome quality to behavioral intention (Est = 0.111, 95% CI [−0.049, 0.261]), and interaction quality to behavioral intention (Est = 0.001, 95% CI [−0.177, 0.173]). The 95% confidence intervals for these estimates included zero, indicating that the direct effects of physical environment quality, outcome quality, and interaction quality on behavioral intention were not statistically significant. These results suggest that satisfaction fully mediates the relationships between these three dimensions of service quality and behavioral intention.

Additionally, the direct path estimate from destination image to behavioral intention was positive (Est = 0.196, 95% CI [0.088, 0.297]), and its confidence interval did not include zero. This indicates that the effect of destination image on behavioral intention was significant through both the direct path and the indirect path mediated by satisfaction. These findings demonstrate a partial mediating effect of satisfaction in the relationship between destination image and behavioral intention. Thus, H1b, H2b, H3b, and H4b were all supported.

### 4.4. Moderating Effects of Sports Event Scale, Tourist Type, and Cultural Context

This study tested the hypothesized moderating effects by comparing the chi-square (χ^2^) values and degrees of freedom (df) between unconstrained and constrained models. The results of the chi-square difference tests for sports event scale (large scale/small scale), tourist type (spectator/athlete), and cultural context (Eastern/Western) are presented in Table 6.

First, the model fit indices for both unconstrained and constrained models, which included the three moderating variables, were found to be acceptable. The chi-square difference test showed significant differences between the unconstrained and constrained models for the sports event scale and cultural context (sports event scale: Δχ^2^ = 37.191, Δdf = 10, *p* < 0.05; cultural context: Δχ^2^ = 16.769, Δdf = 10, *p* < 0.05). This indicates that sports event scale (large scale/small scale) and cultural context (Eastern/Western) have significant moderating effects on the path coefficients. In contrast, the difference between the unconstrained and constrained models for tourist type (spectator/athlete) was not significant (Δχ^2^ = 15.630, Δdf = 10, *p* = 0.111). This suggests that tourist type does not have a significant moderating effect on the path coefficients, leading to the rejection of H7a and H7b.

A detailed examination revealed that physical environment quality and destination image had a stronger influence on sports tourist satisfaction for small-scale sports events than for large-scale events. However, the effects of outcome quality and interaction quality on satisfaction in small-scale sports events were not significant (outcome quality–satisfaction: Est = 0.097, 95% CI [−0.096, 0.285], *p* > 0.05; interaction quality–satisfaction: Est = 0.079, 95% CI [−0.021, 0.290], *p* > 0.05). Therefore, H6b was supported, and H6a was partially supported. Meanwhile, in Western cultural contexts, physical environment quality, outcome quality, and interaction quality had a stronger influence on satisfaction; on the other hand, in Eastern cultural contexts, destination image had a stronger effect on satisfaction. Accordingly, H8a and H8b were supported.

## 5. Discussion

This study conducted a meta-analysis of 39 articles across six databases to examine the structural relationships among sports event service quality, destination image, tourist satisfaction, and behavioral intention. First, the results of the SEM analysis reveal destination image as the most critical factor in enhancing tourist satisfaction. Sports event tourism, as a key component of tourism behavior (Getz, 2008), shares similarities with general tourism behavior in that destination image (e.g., natural environment, cultural attractions, and facility quality) not only shapes tourists’ initial perceptions of the destination but also significantly impacts their satisfaction and behavioral intentions (Prayag & Ryan, 2012; Ramkissoon et al., 2011). Therefore, destination image is considered one of the most important antecedents of tourist satisfaction (Beerli & Martin, 2004). Moreover, considering that destination image has also been linked to emotional bonding, cultural pride, and collective identity, its influence may extend beyond satisfaction to affect tourists’ subjective well-being, particularly in the context of small-scale or community-based sports events.

Next, the physical environment quality, outcome quality, and interaction quality of sports events have a significant impact on tourists’ satisfaction, which in turn influences their future behavioral intentions. The existing literature suggests that the physical environment of a sports event (e.g., stadium facilities, seat comfort, temperature control, lighting, and sound effects) directly affects spectators’ experiences (Slavich et al., 2018). If event organizers provide clean, comfortable, and well-equipped venues for tourists, their experience is likely to be positive, increasing satisfaction (Dash, 2024; Wakefield & Blodgett, 1994). Furthermore, one of the main reasons tourists attend sports events is to enjoy high-quality competitions. When an event meets their expectations with a high-quality performance, their satisfaction increases (Magaz-González et al., 2020; Parasuraman et al., 1988). In particular, the outcome of a competition directly affects satisfaction for fans of a particular team or participants in an event (Cabello-Manrique et al., 2021; Brady & Cronin, 2001). Lastly, interactions between tourists and event staff not only have a direct impact on the tourists’ experience and emotions but also affect their overall perception of an event (Jeong & Kim, 2020). High-quality interactions not only enhance tourists’ experience but also foster emotional bonds between the tourists and the destination, which can lead to positive attitudes and intentions to revisit (Koronios et al., 2019). The synthesis of 39 relevant studies in this research revealed that the influence of each of the three service quality dimensions on tourist satisfaction was similar in magnitude, which contrasts with some previous findings. This result suggests that the three elements of service quality are complementary and collectively contribute to the overall experience, so no single aspect can be overlooked in its importance. Furthermore, these dimensions—particularly physical environment quality and interaction quality—may indirectly contribute to subjective well-being by promoting positive affective experiences, perceived competence, and social connectedness among sport tourists.

Additionally, this study set satisfaction as a mediator and presented the path mechanism of how service quality and destination image affect tourists’ behavioral intentions. The results show that satisfaction fully mediates the effect of the three dimensions of service quality (physical environment quality, outcome quality, and interaction quality) on behavioral intentions. Unlike in previous studies (Milovanović et al., 2021; Shonk et al., 2017), physical environment quality, outcome quality, and interaction quality were found to influence tourists’ behavioral intentions only after enhancing tourists’ satisfaction. Furthermore, satisfaction was found to play an incomplete mediating role in the effect of destination image on tourists’ future behavioral intentions, which is consistent with other studies (Kusumah & Wahyudin, 2024; Chen & Tsai, 2007). This means that destination image influences tourists’ behavioral intentions not only by affecting their satisfaction but also by playing a direct role in the formation of behavioral intentions. Given that tourist satisfaction has been shown to predict positive emotional states and life satisfaction, it is possible that satisfaction with sports events may also serve as a proximate mechanism linking sport tourism experiences to enhanced subjective well-being.

Meanwhile, this study set three moderating variables (event scale, tourist type, and cultural context) to investigate the influence of the three dimensions of service quality and the host destination image on tourist satisfaction under different conditions. The moderating effects of event scale (large scale/small scale) and cultural context (Eastern/Western) were found to be statistically significant, whereas the moderating effect of tourist type (spectators/athletes) was not. Specifically, in terms of the moderating effect of event scale, the impact of physical environment quality and host destination image on tourist satisfaction was found to be greater in small-scale sports events than in large-scale sports events. This is because small-scale events generally rely on limited promotional resources, and their success depends not solely on the sporting competition itself but also on the host destination’s image, local characteristics, and word of mouth among tourists (Jeong et al., 2019). On the other hand, large-scale events tend to rely more on their global transmission channels and media coverage, focusing on the scale and prestige of the event itself rather than the physical environment or local image (Kramareva & Grix, 2021). In small-scale events, due to their limited size and the smaller number of participants, Tourists tend to demonstrate a higher level of engagement in small-scale events and are more sensitive to physical environmental factors such as venue comfort, safety, and cleanliness. These factors directly affect the quality of their experience and emotional responses, thereby significantly enhancing their satisfaction (Jeong & Kim, 2020). In addition, small-scale events often exhibit stronger community attributes and local cultural characteristics. The image of the host destination not only reflects the physical representation of a city or community but also embodies tourists’ psychological projections of culture, sense of belonging, and place identity. Therefore, in the context of small-scale events, effective destination image management strategies and comfortable physical facilities are more likely to translate into increased tourist satisfaction (Fleshman & Kaplanidou, 2023). In contrast, in large-scale sports events, tourists are more likely to focus on the exciting and stimulating aspects of the competition (Jeong & Kim, 2020). Tourists are primarily attracted to large-scale events due to the event’s size, influence, or star appeal. In such contexts, although physical environment quality and destination image still exert some influence, their relative impact is diminished by the “symbolic value” of the event itself (Hassan & Wang, 2024). Therefore, in large-scale events, tourist satisfaction is more likely to be driven by core experiential factors such as the efficiency of event organization, the quality of the competition, and athletic performance (Theodorakis et al., 2013).

Regarding the moderating effect of cultural context, the impact of the three dimensions of sports event service quality (physical environment quality, outcome quality, and interaction quality) on satisfaction was stronger in Western cultures, whereas in Eastern cultures, destination image had a stronger effect on satisfaction. Western cultures are individualistic; on the other hand, Eastern cultures are more collectivistic (Hofstede, 2001), and their cultural background influences individual perceptions and behaviors (Samaha et al., 2014). Western tourists place more emphasis on elements that affect their personal experience. Individual self-fulfillment and subjective experience are highly valued in tourism activities (Biscaia et al., 2023). Tourists tend to focus on whether the services meet their personal needs—for instance, whether the venue facilities are comfortable, the competition is exciting, and the service staff are friendly and efficient. Thus, factors directly influencing the tourist’s experience, such as physical environment quality, outcome quality, and interaction quality, tend to have a greater impact on satisfaction among Western tourists. In contrast, tourists from Eastern cultures typically value social recognition, emphasizing the group’s welfare and social harmony (Hofstede, 2001). Thus, Eastern tourists place more importance on collective experiences than individual experiences. In such a cultural context, the symbolic meaning of the destination image goes far beyond its surface attributes. Elements such as the destination’s culture, history, landscape, and reputation collectively form the core of tourists’ overall experience evaluation, thereby exerting a stronger influence on satisfaction (Ramires et al., 2018), which makes destination image a crucial factor for them. Eastern tourists may place greater importance on whether an event reflects or represents the culture and reputation of the region (Chen & Tsai, 2007). These cultural distinctions also imply that the well-being outcomes of sports event participation may vary by cultural background, warranting future exploration of how satisfaction derived from service quality or destination image translates into SWB across different sociocultural settings.

Regarding the moderating effect of tourist type, the results indicated no significant differences in the effects of host destination image and sports event service quality on satisfaction between different tourist categories, namely spectators and athletes. Based on prior classifications of sports tourists, this study categorized the tourists in the selected literature into two types: spectators, whose primary purpose is to watch the sporting event, and athletes, who participate in the event as competitors (Gibson, 1998). From the perspective of participation motivation, one of the main drivers for athletes is the pursuit of victory. However, this competitive nature may also induce anxiety and psychological stress, potentially leading to negative mental states among athletes (Rice et al., 2016). In contrast, according to the expectancy disconfirmation theory, one of the key motivations for spectators to attend sports events is the enjoyment of an exciting game (Parasuraman et al., 1988). Furthermore, when tourists are highly identified with a specific team or an individual athlete, the outcome of the event may directly affect their satisfaction (Brady & Cronin, 2001). Santos (2013) classified athletes into three subgroups: (1) amateur athletes, (2) semi-professional athletes, and (3) professional/elite athletes. From this categorization perspective, coping with high demands and competitive pressure is crucial to the success of professional athletes, which may also negatively impact their mental well-being (Kuok et al., 2021). Due to their commercial value, professional athletes are often commodified or treated as entertainment assets (Szathmári, 2025), which exposes them to public scrutiny and criticism, further contributing to negative psychological states (Eather et al., 2023). Such effects are not limited to elite athletes—student athletes also experience substantial stress from injury risk and performance expectations, which may adversely affect their satisfaction (Kroshus, 2016). By comparison, amateur athletes tend to report greater psychological benefits and higher satisfaction from their participation in sports events (Eather et al., 2023; Andersen et al., 2019). Although athletes and spectators differ in their roles, both groups contain diverse subtypes. As such, the cognitive and emotional mechanisms through which service quality and destination image influence satisfaction may be similar in some cases and different in others. This ambiguity may have attenuated the moderating effect of tourist type. Overall, tourist type may not function as a standalone moderator; rather, its influence may be conditional upon other contextual factors such as event type or participation motivation. Future studies are encouraged to incorporate these interaction effects and further segment both athlete and spectator subtypes to uncover the nuanced impacts of tourist type on satisfaction and behavioral outcomes.

In conclusion, this research integrated 39 previous studies with 16,335 participants, overcoming the inconsistency between different research results to provide more comprehensive and reliable outcomes. On the one hand, this study validates the applicability of the three-dimensional structure of service quality—physical environment quality, outcome quality, and interaction quality—within the context of sports events, and further supports the explanatory power of expectancy disconfirmation theory in the field of sports tourism, thereby enhancing the external validity of this theory in service experience research. On the other hand, the findings reveal that tourist satisfaction fully mediates the relationship between service quality and behavioral intentions, and partially mediates the relationship between destination image and behavioral intentions, thereby extending the understanding of satisfaction mechanisms within the service marketing literature. In addition, this study incorporates event scale, cultural background, and tourist type as moderating variables, and finds that event scale and cultural context significantly moderate the strength of the structural paths, highlighting the context dependency of behavioral patterns in sports tourism. Although the moderating effect of tourist type was not statistically significant, this study suggests that its influence may be constrained by the specific subtypes of tourists and may interact with factors such as event type and participation motivation. These findings provide theoretical insight for future research to incorporate interaction effects or multilevel analysis models into the study of tourist behavioral intentions. Furthermore, it suggests the potential for future research to explore how sport event experiences contribute to subjective well-being via satisfaction pathways, particularly in post-event contexts.

In practical terms, this study offers valuable guidance for various stakeholders in the sports industry. For event organizers, the findings highlight the crucial role of destination image in enhancing tourist satisfaction, with particularly pronounced effects in the context of small-scale events and Eastern cultural settings. This suggests that organizers should focus on shaping and communicating a cohesive destination image by emphasizing cultural symbols and local characteristics associated with the event, thereby strengthening tourists’ sense of identification and satisfaction. For venue managers and service providers, the three dimensions of service quality—physical environment quality, outcome quality, and interaction quality—were all found to significantly influence tourist satisfaction, especially in Western cultural contexts. Therefore, under the broader agenda of green and sustainable development, attention should be directed toward the maintenance and repair of venue infrastructure and the optimization of service delivery processes, in order to enhance the overall event experience. For athletes and teams, particularly amateur participants, a fair, safe, and appropriately challenging competitive environment not only affects their satisfaction but also contributes to positive psychological experiences and subjective well-being, which may indirectly influence their behavioral intentions and willingness to participate in future events. Finally, the findings can help tourism practitioners accurately identify the key pathways influencing tourist behavioral intentions, enabling them to meet both emotional and functional needs while promoting the integrated development of sports events and tourism resources.

## 6. Conclusions

This study conducted a meta-analysis of 39 articles across six databases, including English and Korean sources, to examine the structural relationships among sports event service quality, destination image, tourist satisfaction, and behavioral intention. Based on this, a structural equation model (SEM) was developed and tested to understand how sports event service quality and destination image influence tourists’ satisfaction and behavioral intentions by synthesizing multiple independent studies. Specifically, first, the host destination image is the most critical factor in enhancing visitor satisfaction. Additionally, the service quality of sports events significantly influences visitor satisfaction, which in turn impacts their future behavioral intentions. Second, tourist satisfaction fully mediates the relationship between event service quality and behavioral intentions, and it partially mediates the relationship between host destination image and behavioral intentions. Third, under the moderating effect of event scale (small scale vs. mega scale), host destination image and physical environment quality are more important in small-scale sports events than in mega-scale sports events. Furthermore, under the moderating effect of cultural context (Eastern vs. Western), service quality dimensions are more influential in Western cultural settings, whereas host destination image is more important in Eastern cultural settings. The significance of this study lies in its integration of previously disparate findings into a unified model, offering a more comprehensive understanding of the relationships among the variables. The results provide broad implications for future academic research and practical insights for sports tourism practitioners.

## 7. Limitations and Future Research Directions

First, this study used all the relevant literature from six major databases, and the results of the review for publication bias and heterogeneity were reliable. However, due to the limited number of studies, the impact of outcome quality and interaction quality on satisfaction in small-scale sports events was statistically insignificant in the test of the moderating effect of event size. Future research could address this limitation by expanding the scope of databases or manually adding studies on small-scale sports events. With regard to the non-significant moderating effect of tourist type, the findings of this study, supported by a review of the relevant literature, suggest that tourist type may not operate as an independent moderating variable. Instead, its influence may only become significant under specific conditions, such as variations in event type or differences in participation motivation. Future research could benefit from incorporating these interaction effects and further segmenting both spectator and athlete categories to better reveal the nuanced and potentially context-dependent impact of tourist type.

Second, this study used the Korean database as a representative source for Eastern countries. Although some English-language studies included research from other Asian countries such as China, Japan, and Indonesia, the analysis did not include databases from these Eastern countries, which is a limitation. Therefore, future studies could obtain more comprehensive results by expanding the data sources. Moreover, since the data included in this study were derived from diverse cultural contexts, event types, and tourist types, the primary purpose of conducting heterogeneity tests was to ensure the appropriateness of the model selection for subsequent SEM analysis and moderator testing. This process also partially accounts for the sources of heterogeneity, which is why a separate heterogeneity source analysis was not conducted. Future research could conduct dedicated analyses of heterogeneity by focusing on potential sources such as differences in measurement tools, sample characteristics, and participation motivations.

Lastly, although the present study did not directly focus on subjective well-being (SWB), the results imply that tourists’ satisfaction with sport event experiences may serve as an indirect mechanism contributing to SWB. Therefore, future research should explicitly examine the relationship between sport event participation and SWB by integrating variables such as life satisfaction, positive affect, and vitality into the structural model. This would allow for a more holistic understanding of the psychological benefits associated with sport event participation.

## Figures and Tables

**Table 1:** The relevant details of the studies included in the analysis.

No.	Study	Method	N.	Related Variables	Tourist Type	Event Name	Country
1	(Ferri et al., 2021)	Correlational	236	IQ, DI, SA, BI	Athlete	Spanish Half Marathon	Spain
2	(Fernández-Martínez et al., 2022)	Correlational	866	PEQ, OQ, SA, BI	Athlete	Spanish 21 km Marathon	Spain
3	(Pahrudin et al., 2024)	Correlational	221	PEQ, IQ, SA, BI	Spectator	MotoGP Grand Prix	Indonesia
4	(Vegara-Ferri et al., 2020)	Correlational	115	PEQ, IQ, DI, BI	Spectator	International Sailing Event	Spain
5	(Elahi et al., 2020)	Correlational	382	DI, SA, BI	Spectator	Iran Pro League (Football)	Iran
6	(Jeong et al., 2019)	Correlational	350	OQ, SA, BI	Athlete	Gyeongju International Marathon	Korea
7	(Quirante-Mañas et al., 2023)	Correlational	866	PEQ, OQ, SA, BI	Athlete	Granada Marathon	Spain
8	(Song et al., 2023)	Correlational	485 459	OQ, BI, DI	Athlete	Xiamen and Chicago Marathon	China USA
9	(Song et al., 2024)	Correlational	313 364	PEQ, OQ, DI, BI	Athlete	Chicago and Xiamen Marathon	USA China
10	(Fernández-Martínez et al., 2021)	Correlational	686	PEQ, OQ, SA, BI	Spectator	European Badminton Championships	Spain
11	(Yamaguchi and Yoshida, 2022)	Correlational	434	PEQ, OQ, IQ, SA, BI	Athlete	Ako City Marathon	Japan
12	S. C. Ma and Kaplanidou (2021)	Correlational	573	PEQ, OQ, IQ, BI	Athlete	Taiwan Marathon	China
13	(Vicente et al., 2021)	Correlational	366	PEQ, IQ, BI	Athlete	Trail Running Competition	Spain
14	(Zhu et al., 2024)	Correlational	702	PEQ, OQ, IQ, SA, BI	Spectator	Esports Tournament	China
15	An and Yamashita (2024)	Correlational	411	PEQ, OQ, IQ, DI	Athlete	Reykjavik Marathon	Iceland
16	(Wang et al., 2021)	Correlational	796	PEQ, OQ, IQ, SA	Spectator	The 66th Macau Grand Prix	China
17	(Leon et al., 2022)	Correlational	384	PEQ, OQ, IQ, BI	Spectator	Esports Tournament	Ecuador
18	(Y. Lee et al., 2019)	Correlational	431	IQ, DI, SA, BI	Spectator	Major Athletics Event	Korea
19	(Xiao et al., 2020)	Correlational	308	PEQ, OQ, IQ, SA, BI	Athlete	Shanghai Marathon	China
20	(Ho Kim et al., 2013)	Correlational	623	PEQ, OQ, IQ, BI	Spectator	College Basketball Game	USA
21	(Yoshida et al., 2013)	Correlational	396	OQ, IQ, BI	Spectator	NCAA Division I College Football Game	USA
22	(K. A. Kim et al., 2019)	Correlational	281	PEQ, IQ, BI	Spectator	Korea Ladies Professional Golf Tour	Korea
23	(S. Lee, 2016)	Correlational	262	PEQ, OQ, IQ, SA	Spectator	Daegu IAAF World Championships	Korea
24	(Kwon et al., 2013)	Correlational	300	PEQ, OQ, IQ, SA, BI	Spectator	Daegu IAAF World Championships	Korea
25	(Min, 2020)	Correlational	396	PEQ, OQ, IQ, DI, SA, BI	Spectator	2019 Gwangju FINA World Championships	Korea
26	Min and Lee (2019)	Correlational	292	PEQ, OQ, DI, BI	Athlete	2017 Tour de Korea	Korea
27	H. Kim and Park (2008)	Correlational	349	PEQ, OQ, DI, SA	Spectator	Women’s Squash World Championships	Korea
28	Min and Woo (2023)	Correlational	217	PEQ, OQ, SA	Athlete	Korea Family Golf Challenge	Korea
29	J. Lee and Kim (2017)	Correlational	286	PEQ, OQ, DI, BI	Athlete	2017 Cheongwon Saengsik Daecheongho Marathon	Korea
30	Seok and Cho (2020)	Correlational	342	PEQ, OQ, IQ, SA	Athlete	International Marathon Championship	Korea
31	(J. H. Park, 2011)	Correlational	222	PEQ, DI, SA	Athlete	National Badminton Championship	Korea
32	J. Kim and Kim (2024)	Correlational	305	PEQ, DI, SA	Athlete	Gangwon Province Youth Winter Olympics	Korea
33	(Ko, 2009)	Correlational	363	PEQ, OQ, IQ, SA, BI	Athlete	The 18th Gyeongju Marathon	Korea
34	(Ji, 2011)	Correlational	389	PEQ, OQ, IQ, SA, BI	Athlete	The 24th Olympic Day Marathon	Korea
35	Oh and Song (2020)	Correlational	339	PEQ, OQ, IQ, DI, BI	Athlete	Regional Marathon Championship	Korea
36	M. Kim and Lee (2018)	Correlational	269	PEQ, OQ, IQ, SA, BI	Spectator	U20 Gyeongju World Cup	Korea
37	J. Park and Park (2015)	Correlational	274	IQ, DI, BI	Spectator	2014 National Elementary School Football Championship	Korea
38	(J. Lee, 2024)	Correlational	300	PEQ, OQ, IQ, SA	Spectator	Korea Ladies Professional Golf Tour	Korea
39	H. Park and Shin (2017)	Correlational	382	PEQ, IQ, SA, BI	Athlete	Ocean Sports Event	Korea

Note. PEQ: physical environment quality, OQ: outcome quality, IQ: interaction quality, DI: host destination image, SA: satisfaction, BI: behavioral intentions, N.: sample size.

**Table 2:** Meta-analysis results.

			Effect Size and 95% Interval				Test of Null (2-Tail)		Heterogeneity			Publication Bias	
Variable	K	N	Correlation		Lower	Upper	Z	*p*	Q(df)	*p*	I^2^	Funnel	Egger’s
PEQ-OQ	26	11,430	F	0.523	0.510	0.537	61.911	<0.001	771.55	<0.001	96.76	S	>0.05
			R	0.530	0.452	0.600	11.257	<0.001	(25)			S	>0.05
PEQ-IQ	22	8546	F	0.582	0.567	0.596	61.245	<0.001	472.37	<0.001	95.55	S	>0.05
			R	0.583	0.512	0.647	12.868	<0.001	(21)			S	>0.05
PEQ-DI	11	3392	F	0.447	0.419	0.474	27.866	<0.001	55.61	<0.001	82.02	S	>0.05
			R	0.448	0.381	0.510	11.715	<0.001	(10)			S	>0.05
PEQ-SA	21	8965	F	0.527	0.512	0.542	55.351	<0.001	641.94	<0.001	96.88	S	>0.05
			R	0.535	0.445	0.614	9.848	<0.001	(20)			S	>0.05
PEQ-BI	24	10,108	F	0.446	0.430	0.461	48.003	<0.001	345.31	<0.001	93.34	S	>0.05
			R	0.455	0.393	0.514	12.570	<0.001	(23)			S	>0.05
OQ-IQ	18	7587	F	0.573	0.558	0.588	56.573	<0.001	714.08	<0.001	97.62	S	>0.05
			R	0.564	0.455	0.656	8.505	<0.001	(17)			S	>0.05
OQ-DI	10	3694	F	0.476	0.450	0.500	31.304	<0.001	30.27	<0.001	70.27	S	>0.05
			R	0.475	0.428	0.520	16.988	<0.001	(9)			S	>0.05
OQ-SA	18	8195	F	0.525	0.509	0.540	52.600	<0.001	946.89	<0.001	98.21	S	>0.05
			R	0.543	0.418	0.648	7.303	<0.001	(17)			S	>0.05
OQ-BI	23	10,443	F	0.462	0.447	0.477	50.967	<0.001	838.19	<0.001	97.38	S	>0.05
			R	0.486	0.390	0.572	8.722	<0.001	(22)			S	>0.05
IQ-DI	7	2202	F	0.511	0.480	0.542	26.363	<0.001	95.09	<0.001	93.69	S	>0.05
			R	0.525	0.391	0.637	6.737	<0.001	(6)			S	>0.05
IQ-SA	16	6121	F	0.532	0.513	0.549	46.172	<0.001	714.86	<0.001	97.90	S	>0.05
			R	0.554	0.421	0.663	6,933	<0.001	(15)			S	>0.05
IQ-BI	21	7772	F	0.434	0.416	0.452	40.834	<0.001	570.32	<0.001	96.55	S	>0.05
			R	0.473	0.374	0.561	8.326	<0.001	(20)			S	>0.05
DI-SA	7	2321	F	0.519	0.489	0.549	27.602	<0.001	39.15	<0.001	84.67	S	>0.05
			R	0.525	0.444	0.597	10.860	<0.001	(6)			S	>0.05
DI-BI	13	4372	F	0.509	0.486	0.530	36.917	<0.001	124.20	<0.001	90.34	S	>0.05
			R	0.520	0.446	0.587	11.693	<0.001	(12)			S	>0.05
SA-BI	14	6198	F	0.637	0.622	0.652	59.129	<0.001	506.82	<0.001	97.44	S	>0.05
			R	0.649	0.549	0.732	9.628	<0.001	(13)			S	>0.05

Note. PEQ: physical environment quality, OQ: outcome quality, IQ: interaction quality, DI: host destination image, SA: satisfaction, BI: behavioral intentions, F: fixed effects model, R: random effects model, S: symmetrical.

**Table 3:** Results of correlations.

	PEQ	OQ	IQ	DI	SA	BI
PEQ	1					
OQ	0.530 ***	1				
IQ	0.583 ***	0.564 ***	1			
DI	0.448 ***	0.475 ***	0.525 ***	1		
SA	0.535 ***	0.543 ***	0.554 ***	0.525 ***	1	
BI	0.455 ***	0.486 ***	0.473 ***	0.520 ***	0.649 ***	1

Note. *** *p* < 0.001. PEQ: physical environment quality, OQ: outcome quality, IQ: interaction quality, DI: host destination image, SA: satisfaction, BI: behavioral intentions.

**Table 4:** Path coefficient.

			95% Confidence Intervals			
Path	Estimates	SE	Lbound	Ubound	z-Value	*p*-Value
PEQ-SA	0.206 ***	0.051	0.107	0.305	4.073	<0.001
OQ-SA	0.207 **	0.065	0.080	0.334	3.194	0.001
IQ-SA	0.198 **	0.068	0.063	0.332	2.886	0.004
DI-SA	0.310 ***	0.043	0.225	0.394	7.191	<0.001
SA-BI	0.758 ***	0.027	0.704	0.812	27.575	<0.001

Note. ** *p* < 0.01. *** *p* < 0.001. PEQ: physical environment quality, OQ: outcome quality, IQ: interaction quality, DI: host destination image, SA: satisfaction, BI: behavioral intentions (model fit indices: simple size = 16335; χ^2^ = 21.208; DF = 4; *p* = 0.0003; RMSEA = 0.016; SRMR = 0.046; TLI = 0.978; CFI = 0.994).

**Table 5:** Mediation effect test.

Path Type		95% Likelihood-Based CIs			Significance
	Path	Lbound	Estimates	Ubound	
Indirect Path	PEQ-SA-BI	0.078	0.156	0.230	Significant
Indirect Path	OQ-SA-BI	0.057	0.157	0.251	Significant
Indirect Path	IQ-SA-BI	0.041	0.150	0.249	Significant
Indirect Path	DI-SA-BI	0.168	0.235	0.298	Significant
Direct Path	PEQ-BI	−0.049	0.074	0.190	Not Significant
Direct Path	OQ-BI	−0.049	0.111	0.261	Not Significant
Direct Path	IQ-BI	−0.177	0.001	0.173	Not Significant
Direct Path	DI-BI	0.088	0.196	0.297	Significant

Note. PEQ: physical environment quality, OQ: outcome quality, IQ: interaction quality, DI: host destination image, SA: satisfaction, BI: behavioral intentions.

**Table 6:** Moderation effect test.

Path	Estimates	Estimates	Free	Constraints	Δχ^2^	Δdf	*p*-Value
Event size	Large scale	Small scale	RMSEA = 0.018	RMSEA = 0.018	37.191	10	<0.05
PEQ-SA	0.189 **	0.233 ***	CFI = 0.995	CFI = 0.988			
OQ-SA	0.229 *	0.097	TLI = 0.981	TLI = 0.980			
IQ-SA	0.256 **	0.079	SRMR = 0.049	SRMR = 0.071			
DI-SA	0.281 ***	0.354 ***	*p* < 0.001	*p* < 0.001			
Tourist	Spectator	Athlete	RMSEA = 0.017	RMSEA = 0.013	15.630	10	0.111
PEQ-SA	0.194 *	0.226 ***	CFI = 0.995	CFI = 0.994			
OQ-SA	0.250 *	0.195 **	TLI = 0.982	TLI = 0.990			
IQ-SA	0.239 *	0.179 **	SRMR = 0.052	SRMR = 0.066			
DI-SA	0.255 **	0.307 ***	*p* = 0.001	*p* = 0.001			
Cultural	Eastern	Western	RMSEA = 0.015	RMSEA = 0.012	16.769	10	<0.05
PEQ-SA	0.159 *	0.311 ***	CFI = 0.997	CFI = 0.996			
OQ-SA	0.186 *	0.223 **	TLI = 0.988	TLI = 0.993			
IQ-SA	0.217 **	0.233 *	SRMR = 0.048	SRMR = 0.058			
DI-SA	0.356 **	0.182 **	*p* = 0.003	*p* = 0.002			

Note. * *p* < 0.05, ** *p* < 0.01, *** *p* < 0.001. PEQ: physical environment quality, OQ: outcome quality, IQ: interaction quality, DI: host destination image, SA: satisfaction, BI: behavioral intentions.

## Data Availability

The original contributions presented in the study are included in the main text and the Appendix A; further inquiries can be directed to the corresponding author.

## Appendix A

**Table A1:** The original relevant information of the included studies.

Study	N	PEQ_ OQ	PEQ_ IQ	PEQ_ DI	PEQ_ SA	PEQ_ BI	OQ_ IQ	OQ_ DI	OQ_ SA	OQ_ BI	IQ_ DI	IQ_ SA	IQ_ BI	DI_ SA	DI_ BI	SA_ BI
(Ferri et al., 2021)	236										0.36	0.29	0.29	0.46	0.46	0.72
(Fernández-Martínez et al., 2022)	866	0.39			0.59	0.53			0.5	0.32						0.66
(Pahrudin et al., 2024)	221		0.75		0.66	0.62						0.73	0.7			0.89
(Vegara-Ferri et al., 2020)	115		0.58	0.59		0.51					0.66		0.76		0.78	
(Elahi et al., 2020)	382													0.52	0.46	0.47
(Jeong et al., 2019)	350								0.69	0.61						0.73
(Quirante-Mañas et al., 2023)	866	0.39			0.59	0.57			0.5	0.44						0.8
(Song et al., 2023)	485							0.52		0.51					0.5	
(Song et al., 2023)	459							0.48		0.34					0.47	
(Song et al., 2024)	313	0.5		0.36		0.5		0.42		0.38					0.4	
(Song et al., 2024)	364	0.41		0.4		0.39		0.44		0.4					0.42	
(Fernández-Martínez et al., 2021)	686	0.32			0.48	0.3			0.44	0.16						0.28
Yamaguchi and Yoshida (2022)	434	0.59	0.66		0.66	0.37	0.55		0.64	0.43		0.56	0.38			0.49
S. C. Ma and Kaplanidou (2021)	573	0.85	0.76			0.62	0.81			0.66			0.53			
(Vicente et al., 2021)	366		0.53			0.5							0.47			
(Zhu et al., 2024)	702	0.43	0.49		0.05	0.06	0.49		0.02	0.01		−0.04	−0.07			
An and Yamashita (2024)	411	0.67	0.51	0.51			0.42	0.42			0.44					
(Wang et al., 2021)	796	0.66	0.6		0.6		0.77		0.73			0.66				
(Leon et al., 2022)	384	0.46	0.3			0.51	0.21			0.48			0.34			
(Y. Lee et al., 2019)	431										0.3	0.4	0.41	0.32	0.41	0.43
(Xiao et al., 2020)	308	0.62	0.78		0.57	0.58	0.63		0.87	0.83		0.6	0.61			
(Ho Kim et al., 2013)	623	0.4	0.65			0.32	0.51			0.79			0.47			
(Yoshida et al., 2013)	396						0.63			0.51			0.22			
(K. A. Kim et al., 2019)	281		0.23			0.19							0.3			
(S. Lee, 2016)	262	0.81	0.84		0.76		0.87		0.8			0.74				
(Kwon et al., 2013)	300	0.57	0.67		0.69	0.57	0.64		0.75	0.65		0.72	0.85			0.7
(Min, 2020)	396	0.79	0.42	0.56	0.83	0.53	0.52	0.45	0.78	0.47	0.72	0.85	0.61	0.6	0.76	0.63
Min and Lee (2019)	292	0.55		0.33		0.39		0.38		0.43					0.46	
H. Kim and Park (2008)	349	0.42		0.3	0.33			0.42	0.38					0.59		
Min and Woo (2023)	217	0.22			0.34				0.42							
J. Lee and Kim (2017)	286	0.55		0.54		0.51		0.58		0.57					0.5	
Seok and Cho (2020)	342	0.29	0.38		0.48		0.01		0.13			0.53				
(J. H. Park, 2011)	222			0.36	0.46									0.58		
J. Kim and Kim (2024)	305			0.36	0.46									0.57		
(Ko, 2009)	363	0.35	0.42		0.42	0.49	0.42		0.37	0.44		0.34	0.36			
(Ji, 2011)	389	0.22	0.46		0.19	0.21	0.2		0.09	0.02		0.24	0.25			0.8
Oh and Song (2020)	339	0.66	0.77	0.57		0.6	0.61	0.61		0.61	0.51		0.56		0.54	
M. Kim and Lee (2018)	269	0.49	0.42		0.48	0.46	0.56		0.55	0.55		0.5	0.49			0.56
J. Park and Park (2015)	274										0.59		0.39		0.47	
H. Park and Shin (2017)	300	0.54	0.54		0.37		0.67		0.31			0.46				
(J. Lee, 2024)	382		0.56		0.58	0.39						0.72	0.48			0.52
